# Correction: Clinical course of Coronavirus Disease-19 in patients with haematological malignancies is characterized by a longer time to respiratory deterioration compared to non-haematological ones: results from a case–control study

**DOI:** 10.1007/s15010-022-01892-x

**Published:** 2022-07-28

**Authors:** A. Oliva, A. Curtolo, L. Volpicelli, F. Cancelli, C. Borrazzo, F. Cogliati Dezza, G. Marcelli, F. Gavaruzzi, S. Di Bari, P. Ricci, O. Turriziani, C. M. Mastroianni, M. Venditti

**Affiliations:** 1 Department of Public Health and Infectious Diseases, Sapienza University of Rome, Piazzale Aldo Moro, 500185 Rome, Italy; 2 Department of Medico-Surgical Sciences and Biotechnologies, Sapienza University of Rome, Rome, Italy; 3 Unit of Emergency Radiology, Department of Radiological, Oncological and Pathological Sciences, Sapienza University of Rome, Rome, Italy; 4 Microbiology and Virology Laboratory, Department of Molecular Medicine, Sapienza University of Rome, Rome, Italy

## Correction: Infection 10.1007/s15010-022-01869-w

In the original version of this article, the given and family names of all authors were incorrectly structured. The corrected author list is given above.

The original article has been corrected.

